# Barriers and Enablers to Adoption of Digital Health Interventions to Support the Implementation of Dietary Guidelines in Early Childhood Education and Care: Cross-Sectional Study

**DOI:** 10.2196/22036

**Published:** 2020-11-20

**Authors:** Alice Grady, Courtney Barnes, Luke Wolfenden, Christophe Lecathelinais, Sze Lin Yoong

**Affiliations:** 1 School of Medicine and Public Health University of Newcastle Callaghan Australia; 2 Population Health Hunter New England Local Health District Wallsend Australia; 3 Hunter Medical Research Institute New Lambton Australia; 4 Priority Research Centre for Health Behaviour University of Newcastle Callaghan Australia

**Keywords:** early childhood education and care, digital health technologies, adoption, dissemination, guidelines

## Abstract

**Background:**

Few Australian childcare centers provide foods consistent with sector dietary guidelines. Digital health technologies are a promising medium to improve the implementation of evidence-based guidelines in the setting. Despite being widely accessible, the population-level impact of such technologies has been limited due to the lack of adoption by end users.

**Objective:**

This study aimed to assess in a national sample of Australian childcare centers (1) intentions to adopt digital health interventions to support the implementation of dietary guidelines, (2) reported barriers and enablers to the adoption of digital health interventions in the setting, and (3) barriers and enablers associated with high intentions to adopt digital health interventions.

**Methods:**

A cross-sectional telephone or online survey was undertaken with 407 childcare centers randomly sampled from a publicly available national register in 2018. Center intentions to adopt new digital health interventions to support dietary guideline implementation in the sector were assessed, in addition to perceived individual, organizational, and contextual factors that may influence adoption based on seven subdomains within the nonadoption, abandonment, scale-up, spread, and sustainability (NASSS) of health and care technologies framework. A multiple-variable linear model was used to identify factors associated with high intentions to adopt digital health interventions.

**Results:**

Findings indicate that 58.9% (229/389) of childcare centers have high intentions to adopt a digital health intervention to support guideline implementation. The *changes needed in team interactions* subdomain scored lowest, which is indicative of a potential barrier (mean 3.52, SD 1.30), with *organization’s capacity to innovate* scoring highest, which is indicative of a potential enabler (mean 5.25, SD 1.00). The two NASSS subdomains of *ease of the adoption decision* (*P*<.001) and *identifying work and individuals involved in implementation* (*P*=.001) were significantly associated with high intentions to adopt digital health interventions.

**Conclusions:**

A substantial proportion of Australian childcare centers have high intentions to adopt new digital health interventions to support dietary guideline implementation. Given evidence of the effectiveness of digital health interventions, these findings suggest that such an intervention may make an important contribution to improving public health nutrition in early childhood.

## Introduction

Poor diet is a modifiable risk factor and leading cause of burden of disease globally [1], with 11 million deaths and 255 million disability-adjusted life years in 2017 attributable to dietary risk factors [2]. Early childhood is a critical period to instill healthy eating habits to reduce diet-related burden of disease, as dietary behaviors developed in childhood track into adulthood [3]. Within Australia, population surveys indicate preschool-aged children are not consuming the recommended servings of fruits and vegetables, and consume more than recommended amounts of discretionary foods (ie, foods high in sodium, saturated fat, and added sugar) [4-6]. As a strategy to reduce the burden from poor diet, the implementation of dietary guidelines in the early childhood education and care (ECEC) setting is recommended [7,8]. Despite such recommendations, Australian childcare centers do not provide foods consistent with sector dietary guidelines [9-11]. For example, a 2017 audit of menus in 70 childcare centers across New South Wales (NSW) determined none of the menus were fully compliant with sector-specific dietary guidelines, particularly for vegetables [9].

Digital health interventions (eg, web-based programs, apps, etc) are advocated by the World Health Organization [12] and offer the opportunity to deliver support at scale and at low cost to improve the nutrition-related practices of food service organizations, such as childcare centers [13]. Evidence from randomized controlled trials [11,14-16] suggests that digital health interventions in education settings can improve the purchasing, provision, and consumption of healthier foods. Despite the clear potential of technology-based approaches, such interventions to improve health outcomes are often not adopted by end users, that is, the individual or organization for which the digital health intervention was developed (eg, ECEC centers, schools, and parents within educational settings). For example, it has been estimated that 80% of health technologies fail [17] due to uncertainty (ie, doubt about the technology’s value or dependability), abandonment (ie, ceasing use of the technology), and lack of organizational willingness to adopt the technology [18] when disseminated in real-world contexts.

Broadly, systematic reviews, guidelines, and previous literature suggest that factors across a number of levels are important for the adoption and implementation of digital health interventions. These include factors related to the individual user (eg, knowledge, skills, beliefs, and attitudes) [19-21], the organization (eg, compatibility or fit with the organization, access to appropriate infrastructure and equipment, and leadership engagement) [12,19-21], the wider setting (eg, external policies and incentives) [12,19,21], the process of implementation (eg, lack of considered planning, engagement, and evaluation) [21], and the technology-based intervention itself (eg, complexity, costs, adaptability, and ability of the intervention to meet user needs) [12,19-21].

Within ECEC settings, a 2015 systematic review examining the barriers to integration of information technology more broadly, including computers, tablets, and touchscreen whiteboards, identified a scarcity of empirical studies examining barriers and enablers within the setting, none of which focused on improving guideline implementation or child health outcomes [19]. The lack of research examining the factors that may enable or impede the adoption of digital health interventions to improve dietary guideline implementation is problematic, as such evidence is necessary to inform future strategies to maximize the adoption and, therefore, impact of evidence-based technologies in the setting.

As such, by employing the nonadoption, abandonment, scale-up, spread, and sustainability (NASSS) of health and care technologies framework [17], this study aimed to describe the following in a randomly selected national sample of Australian childcare centers: (1) intentions to adopt digital health interventions to support childcare center implementation of dietary guidelines, (2) reported individual, organizational, and contextual barriers and enablers to the adoption of digital health interventions in the setting, and (3) barriers and enablers associated with high intentions to adopt digital health interventions.

## Methods

### Study Design, Ethics Approval, and Consent to Participate

This study employed a cross-sectional design. Ethical approval was obtained by the Human Research Ethics Committees of Hunter New England (16/02/17/4.05) and the University of Newcastle (H-2016-0111). All subjects in this research study provided consent to participate.

### Sample

The Australian Children’s Education and Care Quality Authority’s (ACECQA) national register [22] was used to obtain a sampling frame of potentially eligible center-based childcare centers, including long day cares (ie, centers that provide care for children aged 0-6 years for >8 hours per day) and preschools (ie, centers that provide care for children aged 3-6 years for 6-8 hours per day) [5], from each state within Australia (N=10,631). A sample of 1500 childcare centers (14.11%) were randomly selected from the sampling frame of potentially eligible centers, stratified by state and center area socioeconomic classification by an independent statistician.

Childcare center eligibility was assessed via online or telephone survey items. Centers were deemed ineligible if they did not provide meals to children or make menu planning decisions onsite, as this survey was assessing technology to support nutrition guideline implementation on menus; had staff with insufficient English to complete the survey; were a Department of Education and Communities center, as ethical approval was not obtained from the relevant government department; were located in the Hunter New England region of NSW or were select centers across NSW, due to concurrent nutrition and physical activity research trials being undertaken by the research team; were identified as out-of-school hours, vacation care, or family day care; or catered solely to children with special needs.

### Recruitment and Procedures

An email with an information statement and link to an online survey was sent to the nominated supervisor (ie, the center manager) of all sampled childcare centers (N=1500) inviting them to assess eligibility and participate in the study. Nominated supervisors were able to select an alternate staff member (eg, center director) to complete the survey on their behalf. Centers that did not complete the survey within 4 weeks were sent a reminder email to participate (1466/1500, 97.73%), followed by a phone call from a member of the research team (1455/1500, 97.00%) to assess eligibility and gain verbal consent to complete the telephone version of the survey. A final reminder email was sent to centers that indicated a preference to complete the online version of the survey (846/1500, 56.40%) and those who were noncontactable via phone. Centers that were yet to complete the survey following the final reminder email received a final telephone call to gain consent and complete a telephone version of the survey (744/1500, 49.60%). Centers were not offered any incentives to complete the survey. Data to assess study outcomes were collected between January and August 2018.

### Data Collection and Measures

#### Center and Responder Characteristics

Childcare centers were asked to report on the type of center (ie, preschool or long day care), number of full-time equivalent staff members, center opening and closing hours, number of children enrolled, and the number of children enrolled identifying as of Aboriginal and/or Torres Strait Islander background. Childcare center staff completing the survey were asked to report their main role at the center and the total number of years working in the childcare setting. Survey items assessing center characteristics were sourced from previous Australian childcare center surveys conducted by the research team [11,23,24].

Center geographical information, including state and postcode, were obtained via the ACECQA national register to determine location and the center area socioeconomic classification.

#### Intentions to Adopt Digital Health Interventions

To aid comprehension and standardization of digital health interventions and their capabilities, participants were first given a brief example of the potential modality (eg, web-based or online) and key features (eg, feedback and tips) that could be provided within a digital health intervention to support guideline implementation in the setting. Three survey items derived from the Technology Acceptance Model [25] were then used to assess childcare centers’ intentions to adopt digital health interventions in the setting. The Technology Acceptance Model is an information systems theory that models how end users come to accept and use a new technology [25]. The Technology Acceptance Model has been shown to have high internal consistency (Cronbach α>.80) [26]. Respondents were asked to rate on a 7-point Likert scale, ranging from 1 (strongly disagree) to 7 (strongly agree), how much they agreed with the following statements, assuming they had access to a digital health intervention to support the implementation of dietary guidelines in their center: “I intend to use it,” “I predict that I would use it,” and “I would plan to use it.” These items have been used in previous research by the team in the ECEC setting [27].

#### Barriers and Enablers to Adoption of Digital Health Interventions

A purpose-built measure based on the NASSS framework by Greenhalgh [18] was used to assess individual, organizational, and contextual factors that may influence adoption of digital health technologies to improve the implementation of dietary guidelines in the childcare setting. The NASSS framework is an evidence-based, theory-informed, and pragmatic framework designed to help predict and evaluate the success of a technology-supported health care program [18]. The NASSS consists of seven domains: the illness or condition, the technology, the value proposition, the individuals intended to adopt the technology, the organizations, the wider system, and how all these domains interact over time [28]. The NASSS framework can be used to generate insight into the multiple influences on the success or failure of a complex technology-based intervention; to identify simple, complicated, and complex components of the intervention; and to consider how individuals and organizations may be supported to handle complex components of the intervention [18].

An expert advisory group, including health promotion practitioners, implementation scientists, and dietitians, was involved in the development of the measure. Based on expert advisory group consensus, only three of the seven NASSS domains were deemed relevant to the end users for the scale of dissemination of digital health interventions under examination and were, therefore, assessed. At the time of survey development, no validated measure for the NASSS framework existed. As such, a search was conducted for validated measures that had corresponding domains to the NASSS framework. Where possible, such validated measures were employed and adapted to fit the ECEC context, including the e-Health Readiness Measure [29], which was utilized for two of the subdomains: the organization’s capacity to innovate and readiness of the organization for technology-supported change. The e-Health Readiness Measure [30] has been shown to have high internal consistency (Cronbach α>.80). Items for the remaining five subdomains were developed by the advisory group and pilot-tested among a group of health promotion practitioners and trained telephone interviewers for comprehension and face validity. The final measure consisted of 24 items, 10 of which were adapted items from the e-Health Readiness Measure, across three domains and seven subdomains of the NASSS framework, rated on a 7-point Likert scale, ranging from 1 (strongly disagree) to 7 (strongly agree). Table 1 lists the domains, subdomains, number of survey items, and an example survey item relevant to the setting.

**Table 1:** Nonadoption, abandonment, scale-up, spread, and sustainability framework application to the early childhood education and care setting.

Domain and subdomain (No. of items)		Example survey item
**The adopter system**		
	Changes in staff roles, practices, and identities (3 items)	Using an online program is consistent with the usual practices of my cook and menu planner.
**The organization**		
	Organization’s capacity to innovate (6 items)	Overall, I think our service has a champion or leader for using new technology.
	Readiness of the organization for technology-supported change (4 items)	Overall, I think our service has access to experts in use of new technology.
	Ease of the adoption and funding decision (1 item)	It would be easy to adopt new technology to support menu planning in my service.
	Changes needed in team interactions and routines (2 items)	My service would need to change the way it currently plans menus if we decided to adopt new technology.
	Identifying work and individuals involved in implementation (2 items)	We already have the existing personnel available to support the adoption of new technology.
**The wider context**		
	Political, economic, regulatory, professional (eg, medicolegal), and sociocultural context for program rollout (6 items)	I would be more likely to adopt new technology in my service if it was promoted by relevant government agencies (ie, Department of Education or Department of Health).

### Analysis

#### Overview

All analyses were performed in SAS, version 9.3 (SAS Institute) [31]. Descriptive statistics including means, frequencies, and proportions were used to describe center demographic characteristics and survey responses. Childcare center postcodes ranked in the top 50% of NSW, according to the Socio-Economic Indexes for Areas, were classified as *higher socioeconomic status* [32]. A chi-square analysis (ie, test of independence) was used to compare center area socioeconomic classification among consenters and nonconsenters.

#### Intentions to Adopt Digital Health Interventions

An intention-to-adopt score for each responder was calculated by averaging scores for the three intention items. This score was also used to dichotomize responders into having low intentions to adopt (score <6) or high intentions to adopt (score ≥6). This cut point corresponds to those who agree or strongly agree with each item. Such an approach has been used previously within ECEC centers [27].

#### Barriers and Enablers to Adoption of Digital Health Interventions

Similar to previous studies assessing barriers and enablers using theoretical frameworks [9,33-35], average scores for each NASSS construct were calculated by summing all scores for all items within the subdomain, ranging from 1 (strongly disagree) to 7 (strongly agree), and dividing by the total number of responses within the domain. Six survey items were negatively worded and were, therefore, reverse scored. Mean values were used to describe the domains as potential barriers and enablers [33]. A lower mean (≤4) suggested that the particular domain may be a barrier, and a higher mean (>4) suggested a perceived enabler to adoption of digital health interventions. In consultation with the expert advisory group (ie, health promotion practitioners, implementation scientists, and dieticians), this cutoff was employed as a pragmatic approach to categorizing mean scores (ie, ≤4 [responses strongly disagree to neither agree nor disagree] and >4 [responses slightly agree to strongly agree]) and was chosen to limit reporting of any potential social desirability bias in the identification of enablers.

#### Barriers and Enablers Associated With Intentions to Adopt Digital Health Interventions

All seven NASSS subdomains were entered as independent variables into a multiple-variable logistic regression model, to assess which NASSS constructs were significantly associated with high intentions to adopt digital health interventions (ie, dependent variable) after adjusting for each other. The significance value was set at .05.

## Results

### Characteristics of Participants

Of the 1500 centers invited to participate in the study, 72 (4.80%) were noncontactable, 53 (3.53%) were contacted but did not respond, and 378 (25.20%) declined to participate prior to eligibility being assessed. A total of 997 out of 1500 (66.47%) centers consented to the study and were assessed for eligibility, with 590 of these 997 (59.2%) centers deemed ineligible, most commonly due to the center not providing meals and/or snacks to children and being a Department of Education and Community center. This resulted in a total of 407 centers taking part in the survey. There were no statistically significant differences in center socioeconomic area between consenters and nonconsenters.

The large majority of participating centers were long day care centers (391/407, 96.1%) (see Table 2). The majority of responders held the position of nominated supervisor (183/399, 45.9%) or director (179/399, 44.9%), with more than 10 years’ experience working in the childcare setting (278/397, 70.0%).

**Table 2:** Childcare center and responder characteristics.

Characteristics			Value, n (%) or mean (SD)
**Center characteristics (N=407)**			
	**Type of center, n (%)**		
		Preschool	16 (3.9)
		Long day care center	391 (96.1)
	Number of children enrolled (n=406), mean (SD)		96.33 (56.79)
	Number of full-time equivalent primary contact teaching staff (n=404), mean (SD)		12.78 (7.93)
	Number of children of Aboriginal and/or Torres Strait Islander background enrolled at center (n=406), n (%)		214 (52.7)
	**Center area socioeconomic status** **, n (%)**		
		High	231 (56.8)
		Low	176 (43.2)
	**Center geographic location** **, n (%)**		
		Urban (major cities)	307 (75.4)
		Rural (inner regional, outer regional, or remote)	100 (24.6)
	**Center state, n (%)**		
		New South Wales	165 (40.5)
		Victoria	94 (23.1)
		Queensland	62 (15.2)
		Australian Capital Territory	7 (1.7)
		Tasmania	7 (1.7)
		Western Australia	40 (9.8)
		South Australia	25 (6.1)
		Northern Territory	7 (1.7)
**Responder characteristics** **(n=399), n (%)**			
	**Role at the center**		
		Nominated supervisor	183 (45.9)
		Director	179 (44.9)
		Cook	12 (3.0)
		Other	28 (7.0)
	**Number of years working in the childcare setting (n=397), n (%)**		
		≤5	36 (9.1)
		6-10	83 (20.9)
		>10	278 (70.0)

### Intentions to Adopt Digital Health Interventions

The mean intention score was 5.52 (SD 1.07), with a median of 6.00 (IQR 5.00-6.00). Of 389 responders, 229 (58.9%) centers had high intentions to adopt digital health interventions to support the implementation of dietary guidelines.

### Reported Barriers and Enablers to Adoption of Digital Health Interventions

A mean score of 4 or lower (ie, barriers) was found for four of the seven NASSS domains (see Table 3). For three of the seven NASSS constructs—organization’s capacity to innovate, ease of the adoption and funding decision, and political context—responders had mean scores of more than 4 (ie, enablers).

**Table 3:** Mean and median scores for the nonadoption, abandonment, scale-up, spread, and sustainability subdomain barriers and enablers, as reported by responders.

Barrier or enabler		Score^a^	
		Mean (SD)^b^	Median (IQR)
**The adopter system (n=390)**			
	Changes in staff roles, practices, and identities	4.32 (1.25)	4.33 (3.33-5.00)
**The organization**			
	Organization’s capacity to innovate (n=382)	5.25 (1.00)	5.50 (4.67-6.00)
	Readiness of the organization for technology-supported change (n=386)	4.88 (1.03)	5.00 (4.25-5.75)
	Ease of the adoption and funding decision (n=387)	5.22 (1.31)	6.00 (4.00-6.00)
	Changes needed in team interactions and routines (n=389)	3.52 (1.30)	3.50 (2.50-4.00)
	Identifying work and individuals involved in implementation (n=389)	4.35 (1.19)	4.00 (4.00-5.00)
**The wider context** **(n=389)**			
	Political, economic, regulatory, professional (eg, medicolegal), and sociocultural context for program rollout	5.07 (1.08)	5.33 (4.50-6.00)

^a^Constructs are reported on a 7-point Likert scale, ranging from 1 (strongly disagree) to 7 (strongly agree).

^b^A mean of ≤4 suggests that the particular domain may be a barrier; a mean of >4 suggests the domain may be an enabler.

### Barriers and Enablers Associated With Adoption of Digital Health Interventions

Multiple-variable logistic regression analyses revealed a significant association between two of the NASSS subdomains and high intentions to adopt digital health interventions (see Table 4). For every 1-point increase in the *ease of the adoption and funding decision* subdomain, centers were 1.75 times more likely to have high intentions of adopting digital health interventions (95% CI 1.40-2.18; *P<*.001). For every 1-point increase in the *identifying work and individuals involved in implementation* subdomain, centers had 1.46 times the odds of having high intentions to adopt digital health interventions (95% CI 1.61-1.84; *P=*.001).

**Table 4:** Nonadoption, abandonment, scale-up, spread, and sustainability subdomains associated with high intentions to adopt digital health interventions in early childhood education and care centers.

Barrier or enabler		Odds ratio	95% CI	*P* value
**The adopter system**				
	Changes in staff roles, practices, and identities	0.88	0.71-1.10	.27
**The organization**				
	Organization’s capacity to innovate	1.26	0.91-1.75	.17
	Readiness of the organization for technology-supported change	1.15	0.83-1.59	.41
	Ease of the adoption and funding decision	1.75	1.40-2.18	<.001
	Changes needed in team interactions and routines	0.92	0.75-1.13	.42
	Identifying work and individuals involved in implementation	1.46	1.16-1.84	.001
**The wider context**				
	Political, economic, regulatory, professional (eg, medicolegal), and sociocultural context for program rollout	1.03	0.82-1.29	.81

## Discussion

### Principal Findings

This novel study applied a technology-specific framework to conduct a theoretical assessment of childcare center barriers and enablers to the adoption of digital health interventions to improve dietary guideline implementation, nationally. Application of the NASSS framework resulted in the identification of a number of reported barriers and enablers. The main barrier identified was *changes needed in team interactions and routines*, with the main enablers identified as being *ease of the adoption decision*, *identifying work and individuals involved in implementation*, and *organization’s capacity to innovate*. Centers that reported higher scores in the *ease of the adoption decision* and the *identifying work and individuals involved in implementation* subdomains were significantly more likely to have high intentions of adopting digital health interventions.

The study found that over half (229/389, 58.9%) of responders had high intentions to adopt digital health interventions in the setting. Few studies of technology-based health interventions within the ECEC setting report adoption rates, with variable findings. A 2015 cross-sectional study assessing intentions to adopt a web-based program to support healthy eating and physical activity policies and practices in the ECEC setting reported that 72% of respondents had high intentions to adopt such a program [27]. In our earlier study assessing the impact of implementation support on actual adoption of a web-based menu planning program, 58% of the control group, who did not receive support, had adopted the program [36]. Combined, these findings are indicative of the relatively high intentions to adopt digital health technologies in the ECEC setting.

When examining the potential barriers and enablers to adoption of digital health interventions, scores of 4 or higher were found for only three of the subdomains assessed (ie, enablers), two of which fall within the organizational construct of the NASSS framework. The highest levels of agreement were found for the *organization’s capacity to innovate* (mean 5.25, SD 1.00), the *ease of the adoption decision* (mean 5.22, SD 1.31), and *political context* (mean 5.07, SD 1.08). This suggests these subdomains may be potential enablers of the adoption of digital health interventions for end users. Responders reported the lowest level of agreement for *changes needed in team interactions* (mean 3.52, SD 1.30) within the organizational construct, which suggests this subdomain may be a potential barrier to adoption. Such findings suggest that in order to facilitate the adoption of new technology, strategies that generate a high level of organizational support (eg, informing opinion leaders, involving executive boards, and mandating change) and those that overcome any operational challenges and changes in practice (eg, educational outreach visits, changing equipment, and local technical assistance) should be considered [37]. This finding is consistent with previous research demonstrating that implementation support strategies, including face-to-face training, ongoing telephone support, and provision of resources and infrastructure, in addition to obtaining managerial support, improved the adoption of a web-based program in the setting [36].

Study results revealed a discrepancy in the reported barriers and enablers to adoption of digital health interventions and the factors associated with adoption. Multiple-variable logistic regression analyses determined that the *ease of the adoption decision* and the *identifying work and individuals involved in implementation* subdomains were the only factors to have a statistically significant association with high intentions to adopt. Responders scoring higher, that is, those with greater agreement, on these two factors were 1.75 and 1.46 times more likely to report high intentions to adopt digital health interventions, respectively. Although previous studies have not specifically assessed such theoretical constructs in this setting, incongruity in the perceived versus the actual experiences of barriers to the adoption of technology-based interventions [19] and evidence-based guidelines [9] within the ECEC setting has been reported previously. There are opportunities to target this identified incongruence. In-depth examination of the factors by way of supplementation with qualitative methods among all intended end users is warranted. This may provide greater insights into the complexities to adoption of technology-based health interventions and the interaction between each domain of the NASSS.

While recent studies have employed the NASSS framework retrospectively to categorize various constructs [38,39], this study is novel in its prospective application of the NASSS as a measure to conduct a theory-based assessment. Future research could further examine use of the NASSS as a tool to identify barriers and enablers to the adoption of digital health interventions to inform intervention development and evaluation. In addition, embedding measures of the NASSS into the evaluation of dissemination interventions to improve adoption of digital health interventions would allow for an examination of mechanisms and provide a better understanding of how individual, organizational, and contextual factors impact adoption.

### Limitations

The intention to adopt digital health interventions, rather than actual adoption, was assessed. While there is evidence of a relationship between intentions and actual adoption [40], and while our findings align with prior research in the setting [36], rates of actual adoption may differ to those reported. While drawing on validated measures used in other settings, this study employed a nonvalidated self-reported measure to assess barriers and enablers to the adoption of digital health interventions, which may be subject to social desirability bias [41]. Three of the subdomains*—changes needed in team interaction, identifying work and individuals involved in implementation,* and *ease of funding decision*—contained less than three items and, as such, should be interpreted with caution. This study also did not assess additional contextual factors that are theorized to influence adoption according to the NASSS framework, such as the condition, the technology, the value proposition, and embedding and adapting over time [18]. Future studies should consider undertaking an assessment of such factors to assist in providing a more comprehensive understanding of the broader factors that may impact adoption of digital health technologies in the childcare setting. Finally, as Department of Education and Community centers were not eligible to participate, study findings may not be representative of these centers. However, as the geographic distribution of participating centers is similar to that of the sampling frame—all center-based childcare within the ACECQA national register (differences between the responders in each state vs state population ranged from 0.32% to 7.05%)—the sample may be considered nationally representative.

### Conclusions

This study provides novel insights into the perceived and actual factors that may facilitate or impede the adoption of digital health interventions at scale from the perspective of end users. A substantial proportion of Australian childcare centers reported high intentions to adopt digital health interventions. Given evidence of the effectiveness of such technologies, these interventions have the potential to make an important contribution to improving public health nutrition in early childhood. Nonetheless, future efforts to disseminate digital health prevention programs at scale should consider targeting factors within the *ease of the adoption decision* and *identifying work and individuals involved in implementation* subdomains in order for adoption to be ubiquitous in the setting.

## Abbreviations

ACECQA: Australian Children’s Education and Care Quality Authority
CCNSW: Cancer Council NSW
ECEC: early childhood education and care
HNEPH: Hunter New England Population Health
NASSS: nonadoption, abandonment, scale-up, spread, and sustainability
NHMRC: National Health and Medical Research Council
NSW: New South Wales
TAPPC: The Australian Prevention Partnership Centre
